# A three-dimensional measurement method on MR arthrography of the hip to classify femoro-acetabular impingement

**DOI:** 10.1007/s11604-021-01162-0

**Published:** 2021-06-28

**Authors:** Cosimo Nardi, Luisa De Falco, Giuseppe Caracchini, Linda Calistri, Laura Mercatelli, Stefano Cristin, Chiara Lorini, Edoardo Cavigli, Nicholas Landini, Martina Orlandi, Christian Carulli, Vittorio Miele

**Affiliations:** 1 Department of Experimental and Clinical Biomedical Sciences, Radiodiagnostic Unit N. 2, University of Florence-Azienda Ospedaliero-Universitaria Careggi, Largo Brambilla 3, 50134 Florence, Italy; 2 Radiology Unit, Orthopaedic Traumatologic Center, University of Florence-Azienda Ospedaliero-Universitaria Careggi, Largo Palagi 1, 50134 Florence, Italy; 3 Department of Health Science, University of Florence, Viale Morgagni 48, 50134 Florence, Italy; 4 Department of Radiology, University of Florence-Azienda Ospedaliero-Universitaria Careggi, Largo Brambilla 3, 50134 Florence, Italy; 5 Department of Experimental and Clinical Medicine, Division of Rheumatology, University of Florence-Azienda Ospedaliero-Universitaria Careggi, Largo Brambilla 3, 50134 Florence, Italy; 6 Orthopaedic Clinic, Orthopaedic Traumatologic Center, University of Florence-Azienda Ospedaliero-Universitaria Careggi, Largo Palagi 1, 50134 Florence, Italy

**Keywords:** Femoro-acetabular impingement, Hip, Magnetic resonance imaging, Arthrography, Arthroscopy

## Abstract

**Purpose:**

(1) To investigate correlations between different types of FAI and the ratio of acetabular volume (AV) to femoral head volume (FV) on MR arthrography. (2) To assess 2D/3D measurements in identifying different types of FAI by means of cut-off values of AV/FV ratio (AFR).

**Materials and methods:**

Alpha angle, cranial acetabular version, acetabular depth, lateral center edge angle, AV, and FV of 52 hip MR arthrography were measured. ANOVA test correlated different types of FAI with AFR. ROC curves classified FAI by cut-off values of AFR. Accuracy of 2D/3D measurements was calculated.

**Results:**

ANOVA test showed a significant difference of AFR (*p* value < 0.001) among the three types of FAI. The mean values of AFR were 0.64, 0.74, and 0.89 in cam, mixed, and pincer types, respectively. Cut-off values of AFR were 0.70 to distinguish cam types from mixed and pincer types, and 0.79 to distinguish pincer types from cam and mixed types. Cut-off values identified 100%, 73.9%, and 55.6% of pincer, cam, and mixed types. 2D and 3D classifications of FAI showed accuracy of 40.4% and 73.0%.

**Conclusions:**

3D measurements were clearly more accurate than 2D measurements. Distinct cut-off values of AFR discriminated cam types from pincer types and identified pincer types in all cases. Cam and mixed types were not accurately recognized.

## Introduction

Femoro-acetabular impingement (FAI) is a clinical syndrome arising from an abnormal contact of the articular surfaces of the hip, caused by congenital or acquired morphological defects [1]. FAI is more common among young athletes, whose hip cartilage and acetabular labrum suffer from repetitive micro-trauma, giving rise to early osteoarthritis [2]. There are three types of FAI:

- cam type stems from an excessive convexity of the femoral head–neck junction;
- pincer type is due to too wide covering of the femoral head by the acetabulum or acetabular retroversion;
- mixed type has both cam and pincer features and is the most common clinical occurrence [3, 4].

Several two-dimensional (2D) measurement methods are commonly used to identify FAI on radiographs [5]. Some of such methods are also implemented to computed tomography (CT) and magnetic resonance imaging (MRI), including the alpha angle and lateral center edge angle [6, 7]. CT and MRI measurements are adopted in a single slice allowing only a 2D characterization of femoral and acetabular deformities [8]. In the identification of a cam type, the alpha angle varies in relation to X-ray projections and oblique axial or radial CT/MRI views [6, 9]. Furthermore, there is no agreement among authors on which cut-off value of alpha angle should be used [10, 11]. In the identification of a pincer type, the radiologic parameters show poor reliability since they are highly affected by the tilt and position of the pelvis and are not very helpful to quantify acetabular deformities [12]. However, to date, clinical signs supported by some devoted X-ray projections represent the keys parameters on which surgeons decide whether a patient with FAI needs an arthroscopic intervention [10, 13, 14]. Since radiographic signs of FAI are still considered inaccurate and 2D measurement methods run on CT and MRI are suboptimal [15, 16], volumetric measurement methods to classify the type of FAI have been investigated and three-dimensional (3D) measurement software systems for analyzing acetabular diseases have been developed. These tools are already in use for hip dysplasia, they are still relatively unexplored in FAI [17, 18]. Just a few volumetric studies have been done on FAI via CT [19–23]. They measured height, volume, and location of femoral bump as well as femoral head volume to examine impingement points. MR arthrography (MRA) has been carried out on FAI in only one case [24], where a correlation between femoro-acetabular volumes and chondrolabral lesions was found.

On this challenging background, the aim of this retrospective study was to evaluate the role of MRA in identifying different types of FAI, by studying the ratio of acetabular volume (AV) to femoral head volume (FV). The secondary endpoint was to compare 2D vs. 3D methods in the detection of FAI by means of cut-off values of the AV/FV ratio (AFR).

## Materials and methods

### Patients

From January 2014 to December 2019, 119 MRA of the hip were performed in the MR-unit of Careggi Hospital (Florence, Italy), corresponding to 119 patients (only one hip per patient).

**Fig. 1:**
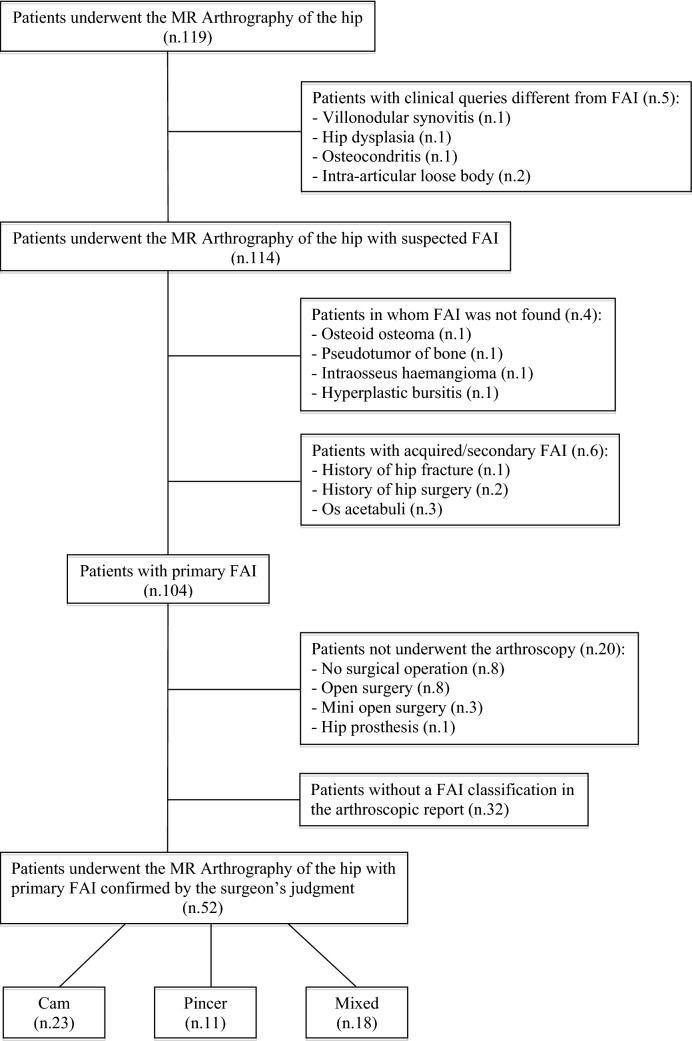
A flowchart of the selection criteria for enrolling patients. Acetabular dysplasia is defined by a lateral center–edge angle < 25°

Exclusion causes were reported in Fig. 1. Fifty-two patients who had undergone the MRA of the hip with primary FAI were enrolled. This study was approved by the research ethics committee (protocol n.27025/2019) and informed written consent was obtained from all patients.

Since no specific intra-operative arthroscopic definition of cam, pincer, and mixed FAI has been suggested in literature [25], the orthopedic surgeon classified the three types of FAI based on an overall judgment including bone deformities on pre-operative hip X-rays, clinical signs [26], intra-operative dynamic examination techniques [27], and chondrolabral abnormalities identified during surgical interventions [3, 28]. He found 23, 11, and 18 cam, pincer, and mixed types, respectively. A correlation between MRA images and the different types of FAI was performed. The analysis of cartilaginous pathologies and labral tears were not investigated being beyond the purposes of our study.

### Device and scan technique

MRA of the hip was performed by a musculoskeletal radiologist with 12 years’ experience (GC). After skin disinfection and the administration of local anesthesia with intramuscular injection of 1% lidocaine hydrochloride solution, the radiologist carried out an ultrasound-guided intra-articular hip injection of 15–20 ml of a solution of gadoterate meglumine (0.0025 mmol/mL) by using a pre-filled syringe for intra-articular use (Dotarem^®^, Guerbet SA, Paris, France).

Scans were performed with the MAGNETOM Aera 1.5 Tesla (Siemens Medical Solution, Erlangen, Germany) and its body coil. T1-weighted sequence was obtained with a spoiled gradient echo 3D associated with DIXON technique for fat saturation (3D GRE T1 VIBE) on coronal plane: echo time 5.93 ms, repetition time 19.0 ms, flip angle 10°, section thickness 0.8 mm with isotropic voxel, field of view 206 × 192 mm, and matrix 238 × 256. Images were reconstructed on axial and oblique planes passing through the center of the femoral neck. Coronal, axial, and axial oblique images were exported (anonymized) in DICOM format and analyzed using OsiriX software (version 7.0; OsiriX, Geneva, Switzerland), implemented in a Macintosh operating system with a 21.5-in monitor (Power Macintosh G3; Apple, Cupertino, Calif).

### Two-dimensional measurements

**Fig. 2:**
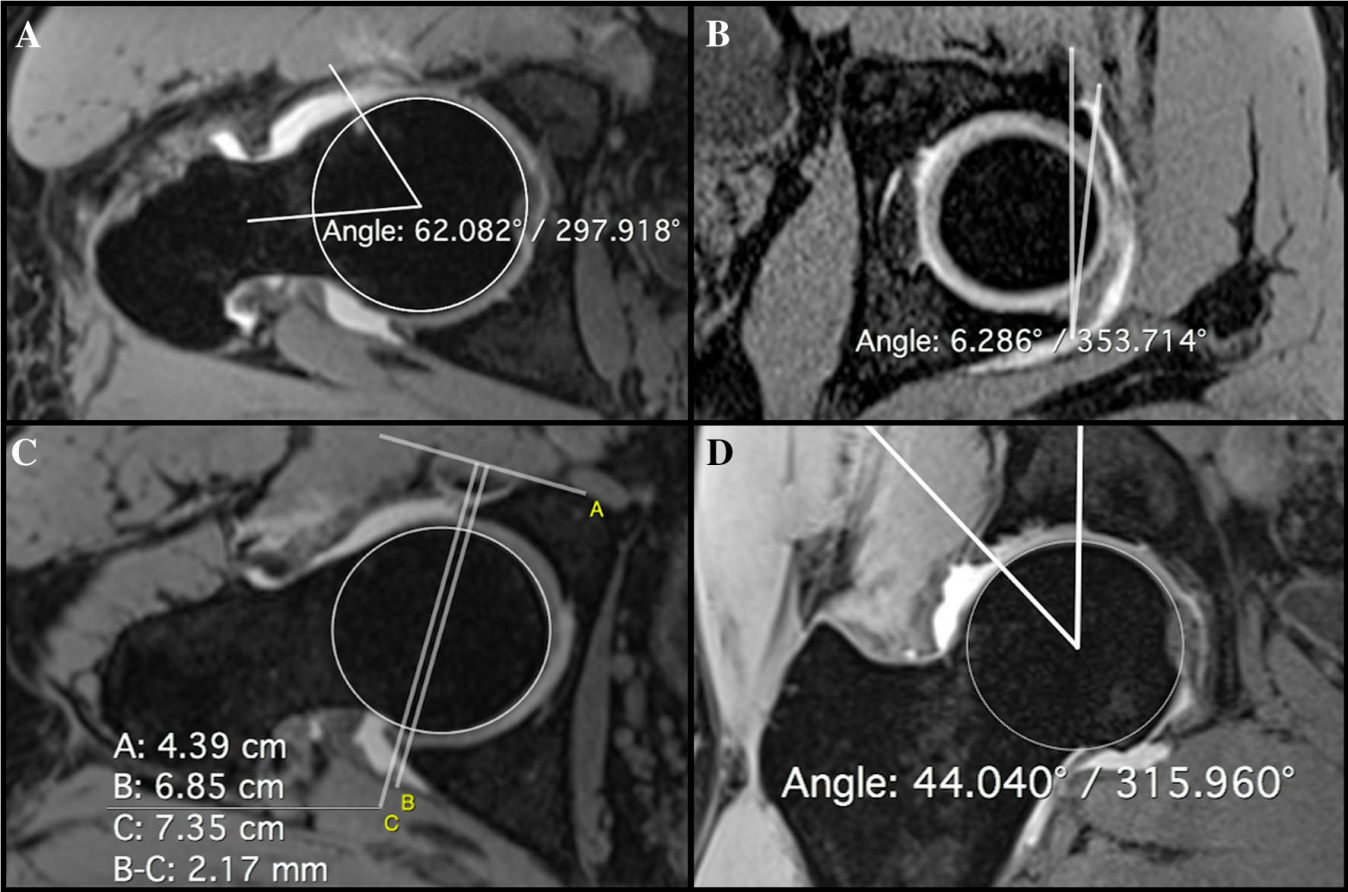
Two-dimensional measurement methods. **A** Alpha angle. Angles ≥ 55° are a sign of cam type FAI. **B** Cranial acetabular version. The angle has to be considered negative (retroversion) when the anterior acetabular margin is lateral to the sagittal plane. Negative values are a sign of pincer type FAI, as the angle of − 6.2° measured in the image. **C** Acetabular depth. Values ≤ 3 mm are a sign of pincer-type FAI. The distance b–c represented in the image is negative (− 2.1 mm) since the center of the femoral head is medial to the line connecting the acetabular rims. **D** Lateral center edge angle. Angles ≥ 40° are a sign of pincer-type FAI

Four 2D measurements were calculated as follows on T1 VIBE images using ROI tool functions named alpha angle, angle, and orthogonal lines (Fig. 2). Osteophytes were excluded from measurements.

1. Alpha angle on the axial oblique images. It was determined by two intersecting lines, the first drawn along the central axis of the neck, the second one from the center of the head to the point where the cortical margin diverged from a best-fit circle surrounding the head [2].
2. Cranial acetabular version on the axial oblique images at the highest point of the acetabulum. It is the angle between the line connecting the anterior and posterior acetabular rims and the sagittal plane. Negative values were defined as cranial acetabular retroversion and positive values as cranial acetabular anteversion [29].
3. Acetabular depth on the axial oblique images. It is the distance between the center of the head—found by using the cross reference with the coronal plane—and the line connecting the anterior to the posterior acetabular rim. The distance had positive values when the center of the head was lateral to the line connecting the acetabular rims. Acetabular depth increased with decreasing measurements [30].
4. Lateral center edge angle on the coronal images. It is the angle between the vertical axis and the line connecting the bone acetabular rim to the center of the head. The center of the head was selected in correlation with the axial oblique plane using a circle as an auxiliary measurement [31].

A patient was classified as follows (Table 1):

- cam type when alpha angle was positive;
- pincer type when at least one parameter between cranial acetabular retroversion, acetabular depth, and lateral center edge angle was positive;
- mixed type when both cam and pincer features were positive;
- negative for disease (healthy person) when no cam and pincer features were detected.

**Table 1:** Two-dimensional measurement values that identified patients affected by femoro-acetabular impingement

2D measurements	Cam type	Pincer type
Alpha angle	≥ 55°	–
Cranial acetabular version	–	≤ 0°
Acetabular depth	–	≤ 3 mm
Lateral center edge angle	–	≥ 40°

### Three-dimensional measurements

**Fig. 3:**
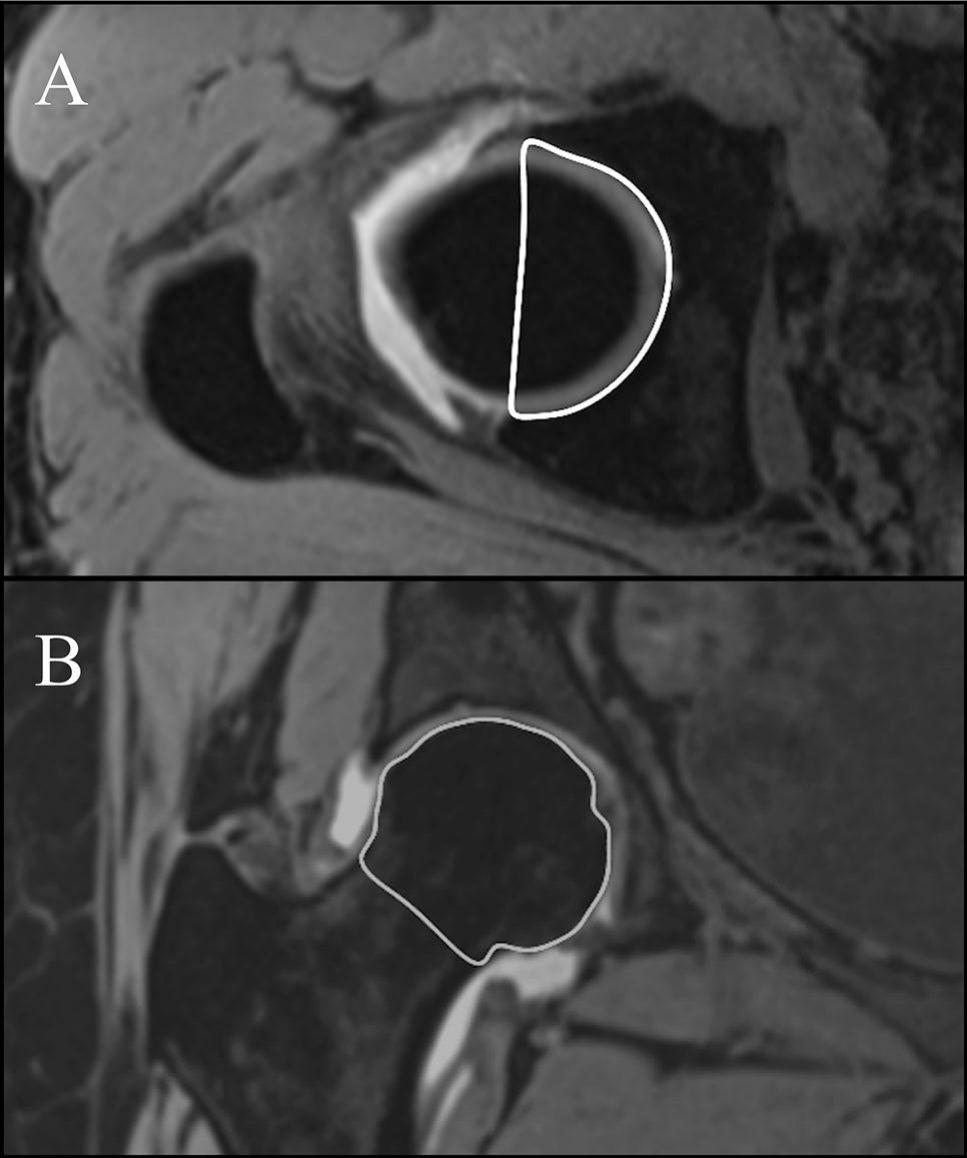
Single-slices volume contouring drawn for 3D measurements. **A** Acetabular cup volume in 2D partition axial plane drawn along the margin of the osseous portion of the acetabular cup. **B** Femoral volume in 2D partition coronal plane drawn along the margin of the femoral head to the narrowest portion of the femoral head–neck junction

All volumes were calculated on T1 VIBE images using the volume ROI function after a free-hand region of interest drawing of the anatomic structures had already been depicted in each slice in which the same structures could be seen (Fig. 3). The volumes were:

- Acetabular volume (AV), calculated on axial images surrounding the acetabular cavity from the top to the plane passing through the transverse ligament and connecting the anterior and posterior acetabular aspects with a straight line.
- Femoral head volume (FV), calculated on coronal images surrounding the head up to the narrowest point of the head–neck junction.

Bone borders were surrounded by manual segmentations; therefore, in measuring AV acetabular cartilage was included and acetabular labrum was excluded, whereas in measuring FV cartilage was excluded. Osteophytes were also excluded from measurements.

To eliminate measurement variability due to different anthropometric parameters of patients, such as gender, height, and physical constitution, AFR of each patient was calculated [32].

### Observers and statistical analysis

All examinations were evaluated by three independent observers (LDF, LM, SC) skilled in musculoskeletal imaging with 7, 5, and 4 years’ experience, respectively. The assessment was carried out twice by each observer—interval of two months—with no prior information about patients. Intra- and inter-observer agreements for 3D measurements were determined by intraclass correlation coefficient (ICC) that it was used to compare the repeatability of several measurements. ICC values of 0.00–0.10, 0.11–0.40, 0.41–0.60, 0.61–0.80, and 0.81–1.0 signified no, slight, fair, good, and very good agreement, respectively. Intra- and inter-observer agreements for the classification obtained by 2D measurement methods were calculated using Cohen kappa. FAI was classified in the three groups (cam, pincer, and mixed types) based on the overall assessment of the three observers (two judgements for each observer). A specific group was set when four out of six judgements were the same, otherwise a discussion was held until the three observers reached a consensus. Kappa values of 0.01–0.20, 0.21–0.40, 0.41–0.60, 0.61–0.80, 0.81–0.99, and 1 represented slight, fair, moderate, substantial, almost perfect, and perfect agreement, respectively.

AFR and the main values of AV and FV were divided in three groups based on the FAI type. The mean, median, and standard deviation values of AV, FV, and AFR were calculated for each group. ANOVA test was used to estimate differences in AFR among the three groups. Multiple comparisons analyses were adopted to check for pairwise significant differences among the groups using the Bonferroni methods. The area under the receiver operating characteristic (ROC) curve was calculated both for AFR and cut-off values of AFR for the three classified groups. A curve was calculated to distinguish a cam type from mixed and pincer types (mixed and pincer types were grouped together). A different curve was also calculated to distinguish a pincer type from cam and mixed types (cam and mixed types were grouped together).

Sensitivity and specificity were calculated for the entire spectrum of values of the best selected variables. Cut-off values were chosen as the values with the highest sensitivity and specificity at the same time. Finally, the agreement between the classifications achieved with cut-off values of AFR and 2D measurement methods was calculated using the Cohen kappa (significant *p* value ≤ 0.05). Collected data were analyzed using the SPSS^®^ v. 25.0 statistical analysis software (IBM Corp., New York, NY; formerly SPSS Inc., Chicago, IL).

## Results

### Agreement among the observers

The intra- and inter-observer agreement was very good for the 3D measurements of the 52 patients (23 cam, 11 pincer, and 18 mixed types) with ICC values of 0.89 and 0.88 both for AV and FV (*p* ≤ 0.05), respectively. The Cohen kappa values for the 2D classifications showed moderate agreement between observer 1 and observer 2 (*K* = 0.48) and between observer 1 and observer 3 (*K* = 0.56). The agreement between observer 2 and 3 was substantial (*K* = 0.61). The intra-observer agreement was substantial for all the observers (*K* = 0.71, 0.72, and 0.78, respectively).

### Cut-off values of the 3D measurement method

ANOVA test showed a significant difference of AFR (*p* < 0.001) among the three types of FAI. Moreover, the multiple comparisons analysis showed significant differences (*p* < 0.001) for each analysis using the Bonferroni methods. The mean AFR values were 0.64 ± 0.09 in cam type, 0.74 ± 0.07 in mixed type, and 0.89 ± 0.10 in pincer type. Therefore, AFR showed a progressively increasing trend from cam type to pincer type and intermediate values in mixed type.

**Fig. 4:**
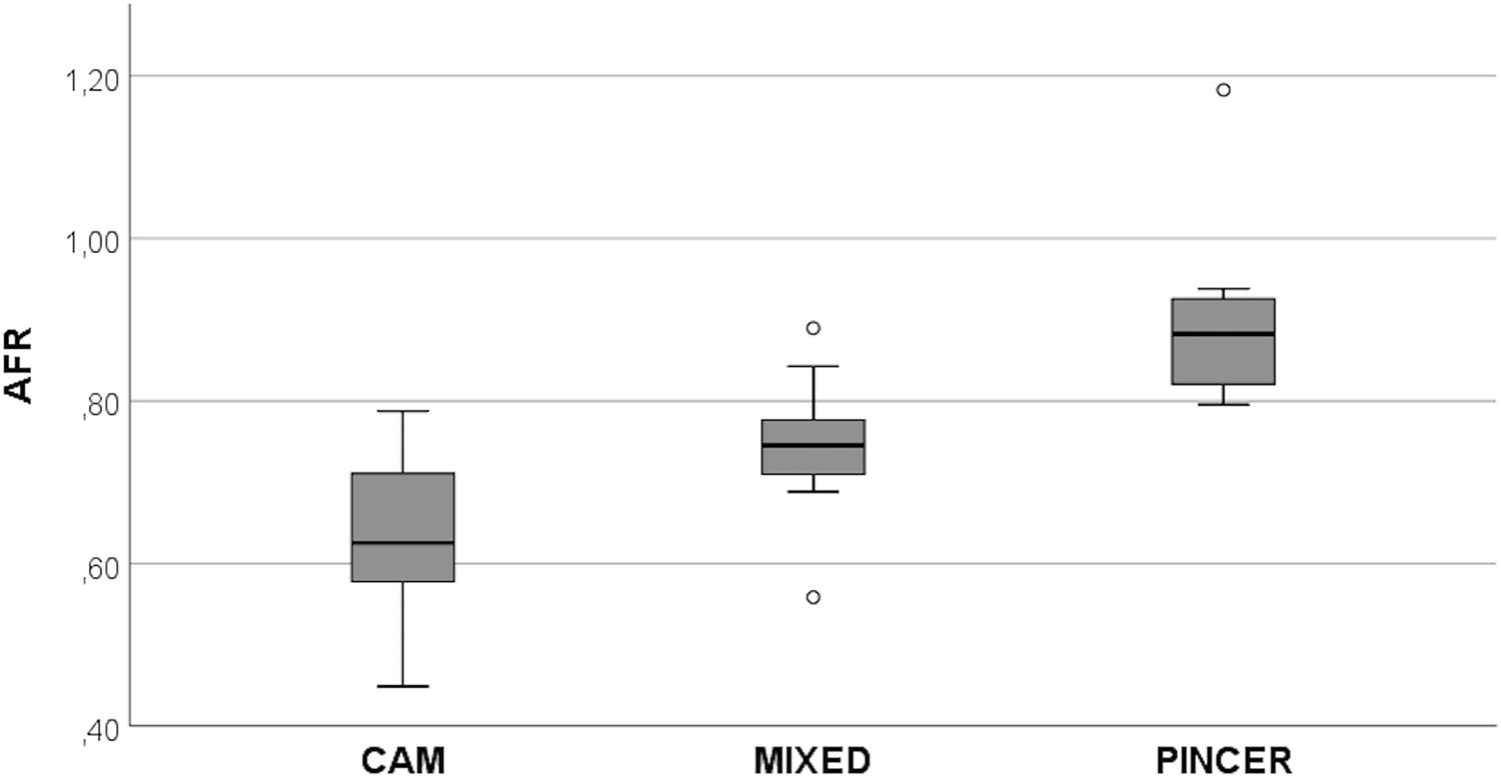
Distribution of acetabular volume/femoral head volume ratio (AFR) in patients affected by femoro-acetabular impingement (FAI). AFR was significantly different among the three types of FAI. It should be noted the overlapping of values between the mixed type and the other two types of FAI (cam and pincer types). No overlapping of values can be observed between cam and pincer types. FAI was represented by 23 cam types, 18 mixed types, and 11 pincer types. ANOVA test: *p* < 0.001. Bonferroni methods for multiple comparisons analysis: *p* < 0.001 for each comparison

**Fig. 5:**
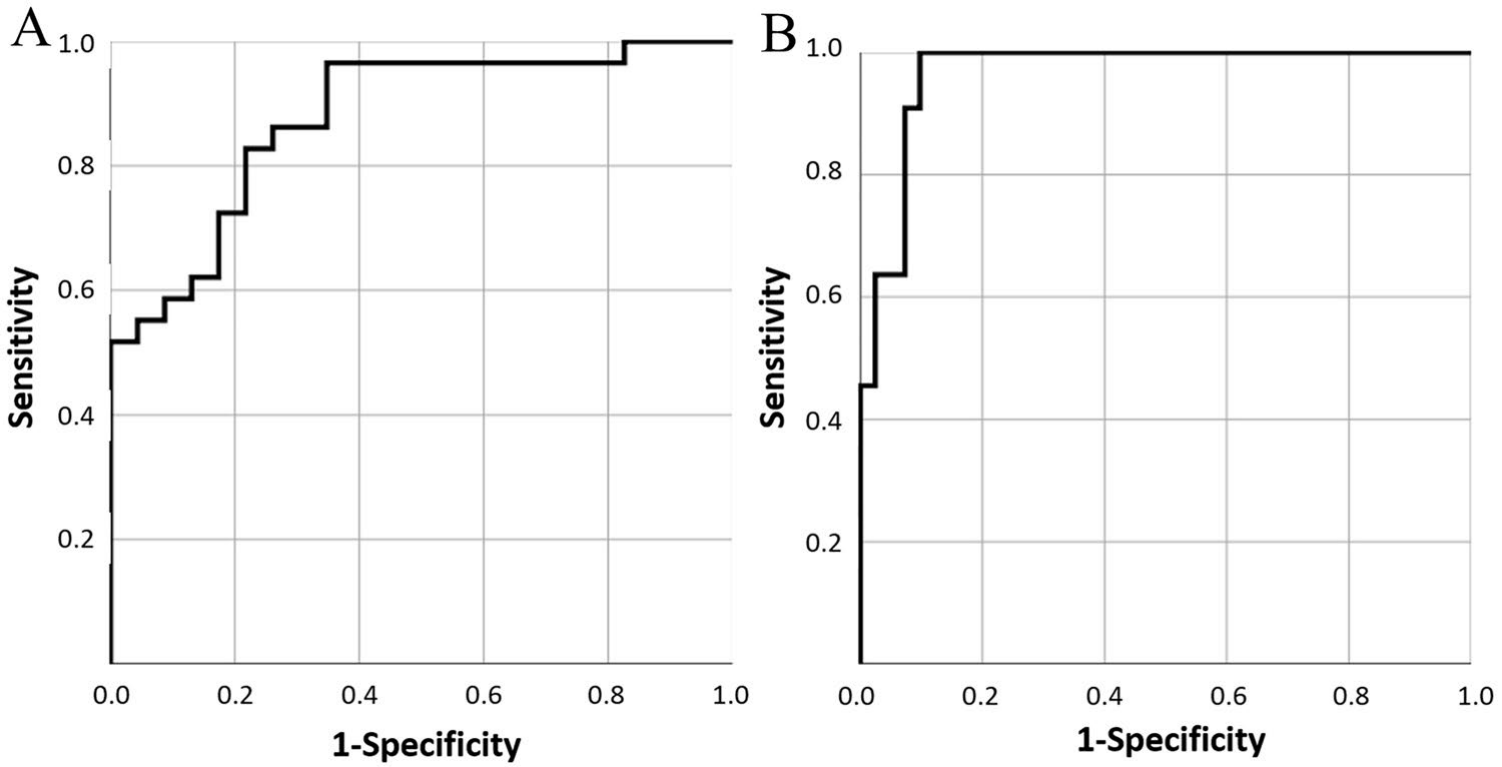
Receiver operating characteristic (ROC) curves showing the accuracy of acetabular volume/femoral head volume ratio (AFR) in discrimination among the three different types of femoro-acetabular impingement. The true positive rate (sensitivity) is plotted in function of the false positive rate (1—specificity). The area under the ROC curve to distinguish a cam type from mixed and pincer types is 0.88 (**A**), and to distinguish a pincer type from cam and mixed types is 0.97 (**B**)

Partial overlap of values between mixed type and cam or pincer types was observed, whereas no overlap was found between cam and pincer types (Fig. 4). The cut-off values of AFR identified by ROC curves were 0.70 to distinguish cam type from mixed and pincer types (sensitivity 86.2%, specificity 73.9%, AUC = 0.877), and 0.79 to distinguish pincer type from cam and mixed types (sensitivity 90.2%, specificity 100%, AUC = 0.967) (Fig. 5).

### Agreement among the surgeon’s opinion on FAI and 2D/3D MRA classification of FAI

At 2D MRA assessment, 44 patients out of 52 resulted positive for FAI with accuracy 40.4%. In these 44 patients the agreement with the surgeon’s judgment was fair (*K* = 0.38) with accuracy 59.1% (Table 2). The remaining 8 patients were deemed as negative for FAI and were equally diagnosed as cam and mixed types at the surgical assessment; 4 out of 8 were correctly identified by the 3D measurement method.

**Table 2:** Contingency tables. 2D classification of patients affected by FAI via MRI arthrogram in relation to the surgeon’s opinion on FAI. (A) Assessment carried out on all the 52 patients. (B) Assessment carried out on only the 44 patients that 2D measurement methods deemed to be diseased

	Cam	Pincer	Mixed	No disease	Total
(A) MRI arthrogram—2D classification (52 patients)					
Surgeon’s opinion on FAI					
Cam	13 (56.6%)	1 (4.3%)	5 (21.7%)	4 (17.4%)	23
Pincer	1 (9.1%)	9 (81.8%)	1 (9.1%)	0 (0%)	11
Mixed	3 (16.7%)	7 (38.9%)	4 (22.2%)	4 (22.2%)	18
Total	17 (32.7%)	17 (32.7%)	10 (19.2%)	8 (15.4%)	52

	Cam	Pincer	Mixed	Total
(B) MRI arthrogram—2D classification (44 patients)				
Surgeon’s opinion on FAI				
Cam	13 (68.4%)	1 (5.3%)	5 (26.3%)	19
Pincer	1 (9.1%)	9 (81.8%)	1 (9.1%)	11
Mixed	3 (21.4%)	7 (50.0%)	4 (28.6%)	14
Total	17 (38.6%)	17 (38.6%)	10 (22.8%)	44

The 3D MRA assessment based on the 52 patients mistakenly classified 6 patients with a cam type as a mixed type, whereas 8 mixed types were mistakenly and equally classified as cam and pincer types. The agreement with the surgeon’s judgment was moderate (*K* = 0.59) with accuracy 73.0%. Considering only the 44 patients positive for FAI by 2D measurements, the agreement was substantial (*K* = 0.66) with accuracy 77.3% (Table 3).

**Table 3:** Contingency tables. 3D classification of patients affected by FAI via MRI arthrogram in relation to the surgeon’s opinion on FAI. (A) Assessment carried out on all the 52 patients. (B) Assessment carried out on only the 44 patients that 2D measurement methods deemed to be diseased

	Cam	Pincer	Mixed	Total
(A) MRI arthrogram—3D classification (52 patients)				
Surgeon’s opinion on FAI				
Cam	17 (73.9%)	0 (0%)	6 (26.1%)	23
Pincer	0 (0%)	11 (100%)	0 (0%)	11
Mixed	4 (22.2%)	4 (22.2%)	10 (55.6%)	18
Total	21 (40.4%)	15 (28.8%)	16 (30.8%)	52
(B) MRI arthrogram—3D classification (44 patients)				
Surgeon’s opinion on FAI				
Cam	14 (73.7%)	0 (0%)	5 (26.3%)	19
Pincer	0 (0%)	11 (100%)	0 (0%)	11
Mixed	1 (7.1%)	4 (28.6%)	9 (64.3%)	14
Total	15 (34.1%)	15 (34.1%)	14 (31.8%)	44

## Discussion

In our series, two cut-off values of AFR were found to discern the different types of FAI. Values < 0.70, from 0.70 to 0.79, and > 0.79 identified cam, mixed, and pincer types, respectively, with no overlap between cam and pincer types. With reference to the surgeon’s opinion, diagnostic concordance of the 3D measurement method was always higher than 2D measurement methods.

Patients with suspicious FAI are examined by 2D measurement methods on X-ray examinations in the first instance [1–5]. Recently, there has been an increased emphasis on 3D software applications and the current study represented the first attempt to design a 3D measurement method able to classify the different types of FAI. The prominence of the femoral head determined a low AFR in cam type (mean value 0.64 ± 0.09), whereas the acetabular overcoverage made AV high with subsequent high AFR in pincer type (mean value 0.89 ± 0.10). In mixed type, the presence of both abnormal features determined an intermediate AFR (mean value 0.74 ± 0.07), thereby overlapping of values between mixed type and cam and pincer types.

Chondrolabral lesions have specific dispositions in relation to FAI types [3, 28]. Kavanagh et al. [24] proved a relationship between AFR and the anatomic area of chondrolabral lesions via hip MRA. They assumed that a low and high AFR corresponded to a cam and pincer type, respectively, even though arthroscopic confirmations were missing. Although the current study did not include labral lesions, it confirmed the deduction of Kavanagh and suggested two cut-off values of AFR to identify the different types of FAI.

The cut-off value of 0.79 discerned a pincer type in 100% of cases. The cut-off value of 0.70 did not assure that this was a cam type but excluded the eventuality of a pincer type for sure. Furthermore, the cut-off values allowed for a classification of mixed type in a little more than half of cases, since AV and FV were variable about the anatomic area—acetabulum or femur—of the predominant bone anomaly. AFR made it possible to recognize whether the predominant anomaly was of an acetabular or femoral nature, thus helping surgical planning. The agreement between diagnosis at the surgery and 3D MRA classification of FAI we have proposed was moderate (*K* = 0.59) but higher than 2D MRA classification (*K* = 0.38). A better agreement was not achieved mainly due to the difficulty in correctly classifying mixed type. Nevertheless, the diagnostic accuracy of the 3D measurement method (73.0%) was higher than 2D ones (40.4%) that incorrectly classified 8 patients as healthy people and in two cases mixed pincer types with cam types. As regards the 44 patients positive for FAI by 2D measurement methods, the diagnostic accuracy of the 3D measurement method further increased (77.3%) and was obviously higher than 2D measurement methods (59.1%).

Being MRA ionizing radiation free, it gives a significant advantage from a protectionist point of view, since people affected by FAI are mainly young adults with a long-life expectancy [33, 34]. However, the obvious invasiveness of the procedure makes MRA a second-level examination that should be recommended in individual cases as a result of clinical and radiographic suspicions [35]. The method proposed in the current study could not only be a useful and efficient complement to MRI arthrogram, but could work alongside or even replace 2D measurement methods used on CT and MRI until now. Further studies will be necessary to strengthen our findings.

Literature reminds us that interpreting a hip X-ray is prone to mistakes due to both the typical disadvantages of 2D imaging in representing 3D structures and the difficulty in positioning patients with hip pain [26]. Even 2D measurement methods implemented on MRI arthrogram are considered suboptimal in FAI assessment [15, 16]. The alpha angle is normally used to diagnose a cam type [7]. Several authors have discussed which alpha angle value should be used as cut-off, ranging between 50° and 60° [10]. Nevertheless, the ability of the alpha angle to discriminate between asymptomatic and symptomatic people is controversial [2]. Appropriate measurements and precise threshold values shall also be defined to identify pincer type [36]. The analysis of acetabulum is especially hard because of its anatomic differences in shape, size, and structure, as well as rim contour irregularities and orientation alterations [37, 38]. Generally, 2D measurement methods focused separately on acetabulum and femur. On the contrary, the 3D measurement method proposed in the present study used a single parameter—AFR—to identify the different types of FAI by specific cut-off values. AFR removed the influence of anthropometric variables and enabled both a simultaneous analysis of femur and acetabulum and an evaluation of interactions between them, the key element of an impingement syndrome. Recently, Fischer et al. [39] demonstrated that alpha angle is associated with age, sex, and anthropometric factors, which have to be taken into account for a better interpretation. The very good agreement between observers with different experience in MRI arthrogram of the hip made the 3D measurement method of the current study easy to reproduce and execute when the clinic query is FAI syndrome. Finding a valid measurement method to classify FAI will not only improve early diagnosis, but even the suitability of treatment planning. An excision to restore the normal concave contour of the femoral head–neck junction and a resection of the prominent acetabular rim are recommended in cam and pincer syndromes, respectively [40]. There is still a long way to go in 3D analysis of femoro-acetabular joint, but our results proved that AFR could play a noteworthy role in the routine study of the hip, not only in case of impingement, but also in dysplasia and micro-instability syndrome. The significant impact of FAI in the development of osteoarthritis has clearly been proved [32, 40]. We emphasized our decision to include patients with osteoarthritis precisely because such disease is an integral part of FAI. Therefore, it is necessary to find a method applicable in clinical practice that can recognize the different types of FAI without excluding patients with osteoarthritis.

A limitation of our study was the unavailability of a control group to discriminate between healthy people and patients affected by FAI, since submitting healthy volunteers to MRA is not ethically justifiable. Reference values of healthy people are known only for 2D measurement methods and not for 3D ones [16, 41]. Several different 2D measurement methods can be used to diagnose FAI, each of these has its own sensitivity and specificity in relation to imaging techniques and X-ray projections [39]. Therefore, we designed our study on MRA images compared to unenhanced MR images to have better anatomic details in measurements. The development of innovative software systems which make 3D measurements accurate and quick to get could facilitate volume calculations in general and especially in unenhanced MR sequences by allowing an easier comparison with healthy people. Manual segmentations of region of interests are difficult, time-consuming, and not always feasible in clinical practice. Thus, intelligent tools are advisable to segment automatically and accurately the boundaries of hip structures [42].

Another limitation was the incalculable nature of femoral versions by 3D measurements. One more weakness was that there were not enough patients for a definite judgment about the feasibility of 3D measurements and cut-off values set. Therefore, the encouraging preliminary results of this study represent a starting point for larger and more detailed studies. This study was performed to determine whether additional researches with big population samples are required or whether there is no role for 3D measurements in FAI. The only one volumetric study that until now analyzed FAI on MR arthrogram images [24] even enrolled lower patients than the current study.

In conclusion, the 3D measurement method on MRA of the hip to classify FAI was clearly more accurate than 2D measurement methods. The 3D measurement method proved that AFR was significantly different among cam, pincer, and mixed types. Distinct cut-off values of AFR enabled both to identify a pincer type and differentiate cam from pincer types, whereas cam and mixed types were not accurately recognized.

## Abbreviations

FAI: Femoro-acetabular impingement
AV: Acetabular volume
FV: Femoral head volume
AFR: Acetabular volume/femoral head volume ratio
CT: Computed tomography
MRI: Magnetic resonance imaging
MRA: Magnetic resonance arthrography
